# Long-Term Neurological Threats of COVID-19: A Call to Update the Thinking About the Outcomes of the Coronavirus Pandemic

**DOI:** 10.3389/fneur.2020.00308

**Published:** 2020-04-17

**Authors:** Antonio Pereira

**Affiliations:** Electrical and Biomedical Engineer Department, Institute of Technology, Federal University of Pará (UFPA), Belém, Brazil

**Keywords:** COVID-19, synucleinopathy, neurotropism, coronavirus (CoV), SARS-CoV

Since 11 March 2020, The World Health Organization has characterized coronavirus disease 2019 (COVID-19), caused by SARS-CoV-2 (1) as a pandemic. The outbreak started in the city of Wuhan in China and quickly spread worldwide. Coronaviruses have previously caused two large-scale pandemics in the past two decades, SARS (2) and Middle East respiratory syndrome (MERS) (3). SARS-CoV-2 is closely related to other CoV found in zoonotic reservoirs, such as bats, camels, and pangolins (4). Since SARS-CoV-2 is considerably more infectious than both SARS-CoV and MERS-CoV, many countries have determined a strict policy of “shelter-in-place” to contain the virus spread through social contagion. However, many questions remain regarding the clinical outcomes of human infection by SARS-CoV-2, including the possibility of the development of neurological disorders (5).

Some viruses possess a tropism for neural tissue and are thus classified as neurotropic (e.g., herpes simplex virus type 1, rabies virus). Those viruses enter the brain through various routes, including retrograde axonal transport along axons, hematogenous spread via the blood-brain barrier (BBB), blood-cerebrospinal fluid barrier, meningeal-cerebrospinal fluid barrier, via direct infection of endothelial cells or through spreading of infected leukocytes to the brain across the BBB (6). Once in the brain, these viruses disrupt the complex organization of neural circuits either directly by neuronal damage or indirectly through host immune response pathways, causing immediate, or delayed neuropathology and neurological manifestations (6) (see below). In the short-term, neurotropic viral infections can cause inflammation of the brain parenchyma and lead to encephalitis or brain-targeted auto-immune responses in susceptible individuals (7). Possible long-term effects on hosts can include alterations on emotional and cognitive behavior, as shown in experimental animals through persistent alterations in the expression of genes involved in the regulation of synaptic activities in key brain areas (8). The axonal transport of neurotropic viruses can also turn intrinsically disordered proteins, such as α-synuclein (α-syn), into promiscuous binders that can form toxic aggregates and travel along neuronal pathways and cause cell death in areas of the brain (9).

While the most common symptoms of COVID-19 at the onset of illness include fever, fatigue, dry cough, myalgia, and dyspnea, other less common symptoms are headache, abdominal pain, diarrhea, nausea, and vomiting (10). Furthermore, it's been recently reported that most patients also complain of impairment of both olfactory and gustatory perception (11) and those are being considered early markers of COVID-19 infection. Though there is longstanding evidence that human coronaviruses, such as SARS-CoV-2, can spread to the brain from the respiratory tract (5, 12, 13), the occurrence of gastrointestinal symptoms (14) suggests that the gastrointestinal system is a possible route of invasion and transmission to the enteric nervous system (ENS) (see Figure 1). While the effects of COVID-2019 on olfactory and gustatory perception may be transient, the possibility that viruses and other contaminant agents can be the initiating etiology of neurodegenerative diseases such as Parkinson's disease (PD) has been raised before (16).

**Figure 1:**
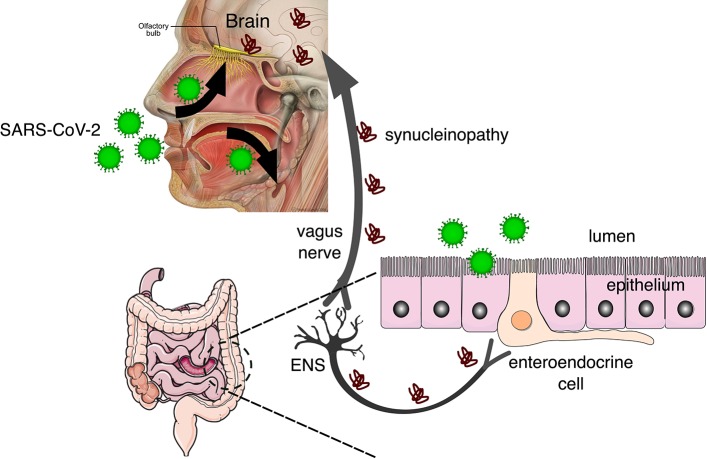
SARS-CoV-2 invades the body through the nasal and oral cavities and may be transmitted to the brain via the olfactory bulb and the enteric nervous system (ENS). In the brain, the virus can cause neuroinflammation by microglial activation and also synucleinopathy that can be transmitted prion-like to other brain regions via the vagus nerve [adapted from Fonseca et al. (15)], from Servier Medical Art, licensed under a Creative Common Attribution 3.0 Generic License (https://www.smart.servier.com/), and Patrick J. Lynch, licensed under a Creative Common Attribution 2.5 License (https://commons.wikimedia.org/wiki/File:Head_olfactory_nerve_-_olfactory_bulb_en.png).

Parkinson's disease (PD) is a common neurodegenerative disorder associated with the progressive loss of dopaminergic neurons located in the midbrain nucleus substantia nigra pars compacta (SNpc) due to the accumulation of α-synuclein (α-syn) aggregates. The Braak hypothesis (9) for the etiology of sporadic Parkinson's disease (PD) proposes that a neurotropic virus invading neural tissue through the nasal cavity and the gastrointestinal tract causes α-syn to turn into a promiscuous binder and be transmitted, prion-like, to key areas such as the SNpc (15). Interestingly, the prodromal or preclinical phase of PD is also characterized by olfactory and gastrointestinal symptoms (17).

The cellular receptor for SARS-CoV-2 is the angiotensin-converting enzyme 2 (ACE2), which has a role in the metabolism of angiotensin peptides involved in the control of vasoconstriction and blood pressure (18). ACE2 is found in several tissues associated with cardiovascular function, but also in the brain, including brainstem nuclei involved with cardio-respiratory regulation (19, 20). Thus, respiratory problems in COVID-19 patients could also derive from the direct action of SARS-CoV-2 in respiratory control nuclei in the Brain (21). Through its binding to ACE2 receptors, SARS-CoV-2 may spread transneuronally to distant brain targets, similar to other neurotropic viruses (22), as predicted by the Braak hypothesis.

Thus, recovery may be an ambiguous term regarding COVID-19. Though recovery from the acute phase of the infections is certainly a relief in public health terms, helping to stop the spreading of the infection, one must consider the long-term neurological effects of the disease. This discussion has been conspicuously lacking in pertinent forums and needs to be adequately addressed as an important concern by public health officials. Many authorities are focusing only on the risks posed to the elderly and immunocompromised subjects, downplaying the threats to younger populations. Though the neurological risks described in the present work are particularly important to the elderly, due to age-related degenerative processes in the immunologic system and the brain, the population needs to be alerted to the chronic neurological risks during the pandemic and maintain social distancing for as long as it is necessary.

## Author Contributions

AP conceived and wrote the manuscript.

### Conflict of Interest

The author declares that the research was conducted in the absence of any commercial or financial relationships that could be construed as a potential conflict of interest.
